# Screening and preclinical assessment of novel *Mycobacterium tuberculosis* recombinant antigens based tuberculin skin testing

**DOI:** 10.3389/fimmu.2025.1498448

**Published:** 2025-03-07

**Authors:** Xichao Ou, Eryong Liu, Faiqa Rashid, Shaojun Pei, Guoqin Zhang, Richard Anthony, Ruida Xing, Fei Huang, Hui Xia, Yang Zheng, Yuanyuan Song, Yang Zhou, Bing Zhao, Shengfen Wang, Minggui Lin, Lin Zhou, Yanlin Zhao

**Affiliations:** ^1^ National Key Laboratory of Intelligent Tracking and Forecasting for Infectious Diseases, Chinese Center for Disease Control and Prevention, Beijing, China; ^2^ National Center for Tuberculosis Control and Prevention, Chinese Centre for Disease Control and Prevention, Beijing, China; ^3^ Department of Bioinformatics and Biosciences, Capital University of Science and Technology, Islamabad, Pakistan; ^4^ School of Public Health, Peking University, Beijing, China; ^5^ Department for Tuberculosis Control Research, Tianjin Center for Tuberculosis Control, Tianjin, China; ^6^ National Tuberculosis Reference Laboratory, National Institute for Public Health and the Environment, Bilthoven, Netherlands; ^7^ Beijing Tsinghua Changgung Hospital, School of Clinical Medicine, Tsinghua University, Beijing, China

**Keywords:** MTB recombinant antigens, tuberculin skin test, screening, preclinical efficacy, EM

## Abstract

**Introduction:**

A new class of *Mycobacterium tuberculosis* (MTB) antigen-based skin tests was recommended by WHO for the diagnosis of TB infection. However, their performance in some settings remains suboptimal. Our study focused on screening novel MTB recombinant antigens for skin tests and evaluating their preclinical efficacy for TB infection detection.

**Methods:**

We constructed *Escherichia coli* BL21 expression vectors to produce a series of recombinant MTB antigens. We assessed their ability to detect tuberculosis infection through skin tests. Model animals sensitized with MTB and BCG were treated with a total of 24 MTB recombinant antigens.

**Results:**

Out of 24 tested recombinant MTB antigens, only three (E-M, E-C-M, and E-7.7-C) met the criteria for skin test reaction interpretation for preclinical trials. Among these, the mean values of skin reaction from E-M were found to be comparable to EC (P>0.05), with no cross-reaction with BCG. Additionally, E-M exhibited a strong safety preclinical profile with no significant abnormalities in physiological, biochemical, or histopathological assessments, supporting its suitability for clinical evaluation. It also displayed high specificity by differentiating MTB infection from BCG vaccination and NTM infection, with no cross-reactivity observed in sensitized guinea pigs.

**Discussion:**

Our results indicate that the E-M recombinant antigen possesses promising characteristics for the detection of tuberculosis infection, demonstrating good safety and efficacy at the preclinical level. Further clinical trials are required to assess its clinical safety, efficacy, and feasibility.

## 1 Introduction

Tuberculosis (TB) is a worldwide health hazard, caused by the *Mycobacterium tuberculosis* (MTB) (1). The ultimate goal of World Health Organization (WHO) End TB Strategy is to control the International TB epidemic by 2035, through decreasing global TB occurrence and death rates by 90% and 95%, respectively (2–4). To meet this goal, WHO recommended the tuberculin skin test (TST) as well as interferon gamma release assay (IGRA) to detect Latent TB Infection (LTBI) which accounts for one quarter of TB burden round the globe (5, 6). Although IGRAs have higher specificity than TST, their high cost, operational complexity, demanding specialized equipment and expertise limit their potential especially in resource-constrained settings. Hence, TSTs remain more practical and the preferred choice for massive TB screening compared to IGRAs in spite of their drawbacks. In 2022, TBST (antigen-based skin test) was authorized by WHO as a screening method for tuberculosis bacillus infection (7–9). This approach replaced conventional TSTs composed of a complicated and diverse mixture of antigenic components, some with significant homology to antigens from BCG and NTM while a small number of more specific MTB antigens.

Currently, there are three globally marketed products based on the TBST approach: C-Tb from India, Diaskins test from Russia and recombinant tubercle bacillus fusion protein (EC) from China (10, 11). These products specifically use the ESAT-6 and CFP-10 proteins from the RD1 region of MTB. Several publications suggested that proteins PE35, PPE68 and EspJ in RD1 region of recombinant MTB can also serve as *in vitro* stimulating antigens for IL-2 or IFN-γ release assays for active tuberculosis diagnosis (12, 13). The recombinant MPT64 protein encoded by *Rv1980c* was found only in MTB and has been utilized for both IFN-γ release testing and inducing delayed-type hypersensitivity (DTH) in guinea pigs with infection (14, 15). The antigenic peptide segment Rv3425 (PPE57) from the RD11 region can stimulate IFN-γ secretion in peripheral blood from tuberculosis patients, but cross-reacts with BCG recipients (16). Antigen based tuberculosis DNA vaccines demonstrate good immunogenicity (17). TB7.7 serves as one of the stimulating antigens in the QuantiFERON-TB Gold In-Tube test (QFT-GIT) for IGRA diagnosis (18). HspX is another protein linked with latent infection and has been utilized for the serological diagnosis of tuberculosis infection (19). DPPD skin tests have shown superior diagnostic performance in HIV/TB co-infected patients compared to TB-PPD (20). Therefore, we hypothesized that PE35, PPE68, EspJ, MPT64, PPE57, TB7.7, HspX, and DPPD, like ESAT-6 and CFP-10, might hold potential value for infection via skin tests.

Therefore, the present study aimed to elucidate the distinct diagnostic potential of the recombinant MTB antigens produced in *Escherichia coli.* We assessed skin test responses in guinea pigs sensitized with MTB or Bacillus Calmette-Guérin (BCG) to identify suitable recombinant antigens for tuberculin skin tests, with commercially available EC serving as a control. Furthermore, the respective recombinant MTB antigens underwent evaluation for preclinical trials and safety establishing them as reference points for future clinical research.

## 2 Materials and methods

### 2.1 Ethical approval

The current study was approved by Ethical Committee of Chinese Center for Disease Control and Prevention with issue no. 2022-CCDC-IACUC-011.

### 2.2 Animal models

Hartly guinea pigs for skin tests were sourced from the Animal Center of China National Institutes for Food and Drug Control. SD rats and guinea pigs designated for active systemic allergic reactions were acquired from Beijing Charles River Experimental Animal Tech Co., Ltd. New Zealand rabbits were procured from the Beijing Fuhao Experimental Animal Breeding Center.

### 2.3 Preparation of recombinant antigen and their grouping with fusion proteins

The recombinant MTB antigens used in this study were supplied by A&B Biotechnology Limited. briefly, the DNA sequence encoding *ESAT6-MPT64* was cloned into the pET28A-SUMO vector to generate pET28A-*SUMO-EAST6-MPT64*. This construct was transformed into *E. coli BL21* (DE3) for protein expression. Transformed cells were cultured in LB medium at 37°C until the OD_600_ reached 0.4–0.5, followed by induction with 0.25 mmol/L IPTG and incubation for an additional 3 hours at 37°C. The His-tagged SUMO-ESAT6-MPT64 fusion protein was purified using Ni-NTA resin. The eluted protein was then treated with His-tagged SUMO protease to remove the HIS-SUMO tag. The cleavage reaction mixture was passed through the Ni-NTA column again, and the untagged E-M protein was collected from the flow-through. The final products were buffer exchanged in PBS. Other untagged recombinant MTB proteins were produced using a similar protocol. For His-tagged protein production, the pET28B vector was used, followed by a similar expression and purification process, omitting the tag removal step. Group antigens included E 、 C 、 M 、 E-M 、 E-C-M 、 E-M-C 、 E-P57, group B: 7.7 、 E-7.7 、 C- 7.7 、 E-C-7.7 、 E-7.7-C 、 7.7-E-C 、 D, E-C-D while group C: P35 、 P68 、 E-P35 、 E-P68 、 E-P35-C 、 E-P68-C 、 E-EJ 、 E-H 、 E-C-H. SDS-PAGE gel in **Supplementary Material S4**, demonstrating that all purified proteins exhibit a predominant single band at the expected molecular weight.

### 2.4 Sensitization of the guinea pigs

Sensitization of guinea pigs for antigen selection: TB-PPD negative guinea pigs were divided into two groups. The MTB sensitization group was designed to evaluate the efficacy of recombinant proteins in detecting TB infections, while the BCG sensitization group ensured that these proteins did not exhibit cross-reactivity with BCG. In the MTB sensitization group, each guinea pig was subcutaneously injected with 0.2 ml of live MTB (50mg/ml, approximately 2.5 x10^9^CFU/ml) in the groin area. In the BCG sensitization group, each guinea pig was subcutaneously injected with 0.2 ml of live BCG (50mg/ml, approximately 2.5 x10^9^CFU/ml) in the groin area. A booster was administered 2-3 weeks post-initial sensitization followed by skin tests conducted 4-5 weeks after the initial sensitization.

Sensitization of guinea pigs for specificity testing: TB-PPD negative guinea pigs were divided into 13 groups, each containing five animals. They were sensitized subcutaneously with 0.2 ml of either MTB, inactivated MTB, BCG-1331, BCG-D2 and nine Mycobacterium species (**Table 1**) in the groin. A second sensitization was performed 2-3 weeks following the initial one, with skin tests carried out 4-5 weeks after the first sensitization.

**Table 1:** Results of 24-hour skin test reactions in guinea pigs sensitized with different sensitizing strains.

Sensitizing strain	Skin test reagent and concentration					
	EC 50U/ml	TB-PPD 50IU/ml	PPD-B 5μg/ml	EM 5μg/ml	EM 10μg/ml	NTM-PPD 5μg/ml
M.tb	5/5	5/5	ND	5/5	5/5	ND
Inactivated M.tb	0/5	5/5	4/5	0/5	0/5	ND
BCG-1331	0/5	5/5	5/5	0/5	0/5	ND
BCG-D2^a^	0/4	4/4	4/4	0/4	0/4	ND
*Mycobacterium kansasii*	3/5	3/5	1/5	1/5	2/5	5/5
*Mycobacterium fortuitum*	0/5	0/5	1/5	0/5	0/5	5/5
*Mycobacterium simiae^a^*	0/4	1/4	4/4	0/4	0/4	4/4
*Mycobacterium gordonae*	0/5	0/5	3/5	0/5	0/5	5/5
*Mycobacterium scrofulaceum*	0/5	0/5	5/5	0/5	0/5	5/5
*Mycobacterium intracellulare*	0/5	1/5	5/5	0/5	0/5	5/5
*Mycobacterium avium*	0/5	0/5	5/5	0/5	0/5	5/5
*Mycobacterium chelonae*	0/5	0/5	4/5	0/5	0/5	3/5
*Mycobacterium abscessus*	0/5	0/5	2/5	0/5	0/5	4/5

^a^One sensitized guinea pig died, leaving a total of four guinea pigs for the skin tests. Data in the table: The numerator represents the number of guinea pigs showing positive reactions, and the denominator represents the total number of guinea pigs tested. ND, not done.

To sensitize guinea pigs for studying skin test characteristics and evaluating the potency of stock solution, TB-PPD negative guinea pigs were injected bilaterally in the groin parts of the hind legs with 0.2 ml of attenuated MTB strain H37Ra (5mg/ml), and skin tests were performed 4-5 weeks post-sensitization.

### 2.5 Guinea pig skin tests

Primary skin test round: Recombinant MTB antigens were divided into three groups. Each group was tested on at least four guinea pigs sensitized with MTB and at least four guinea pigs sensitized with BCG. Each animal received intradermal injections of 0.1ml of the corresponding recombinant antigen (1μg), in addition to 0.1ml each of EC and TB-PPD. Observations of skin erythema or the diameter of induration/erythema (both longitudinal and transverse diameters in mm, with the larger response of either induration or erythema being recorded) were recorded 24- and 48-hours post-injection.

Secondary skin test round: Antigens shortlisted from the first round were tested on at least four guinea pigs previously sensitized with MTB. All subjects received intradermal injections of 0.1ml with 0.5μg of the selected antigen, alongside controls of 0.1ml each of EC and TB-PPD. Observations of skin erythema or the diameter of induration/erythema were recorded as recorded in the first round.

Skin test for specificity assessment: Guinea pigs shed off their hairs dorsally on sensitization with various strains. Later, they received intradermal injections of 0.1 ml of TB-PPD, EC, intracellular mycobacterial pure protein derivative (PPD-B), EM and the pure protein derivative from NTM-sensitized strains (NTM-PPD). Observations of skin erythema or the diameter of induration/erythema were collected by following the steps of first round.

Skin test for characterization of EM-induced skin reactions: At least six guinea pigs sensitized with MTB were prepared by dorsal hair removal. They were administered intradermal injections of 0.1 ml of either 50IU/ml TB-PPD, 50U/ml EC, or EM at concentrations of 2.5μg/ml, 5μg/ml, and 10μg/ml. Four of these guinea pigs were monitored every two hours post-injection for localized indurations or erythematous reactions. Once observed, monitored for every 24 hours until these reactions diminished. Additionally, 24 hours post-injection, two guinea pigs were euthanized, with skin tissues from the injection sites harvested and fixed in formaldehyde for histopathological examination.

### 2.6 Criteria for skin test reactions’ interpretation

- Reactions with an average dimension (considering both axes) of ≥5mm for either induration or erythema were prioritized and declared as positive.
- In contrast, those with dimensions <5mm were categorized as negative.

### 2.7 Clinical observations

All animals were observed twice daily (morning and the afternoon, for mortalities and clinical manifestations. Primary test group animals were thoroughly clinically examined one day prior to the first dose and weekly onwards when removed from their cages. Animals were observed for skin and visible mucous membranes, neurological in addition to behavioral activity, digestive reactions, respiratory presentations, and signs from the urinary and reproductive tracts. The injection site was scrutinized for reactions such as erythema, congestion, swelling, ulceration and induration on the day before administration and the day after, with weekly checks thereafter.

### 2.8 Weighing

All animals were weighed prior to the administration, 2^nd^, 4^th^, and 7^th^ day of the initial dosing, subsequently every week, and before the scheduled euthanasia.

### 2.9 Body temperature measurement

Rectal temperature was measured for the primary test group of animals one day prior to the initial dosing, 2-5 hours post the first dosing (D1), on the second day after the first dose (D2), 2-5 hours post the last dosing (D15), on the second day after the last dose (D16), and before the end of the recovery period (D31) for last 10 subjects each (balanced between male and female) from the high-dose group, low-dose group, and the solvent control group.

### 2.10 Blood cell count, coagulation function, and blood biochemistry analysis

During the administration period, blood was drawn from the jugular vein of the primary test group (last 5 subjects per gender per group) for blood cell counts and fibrinogen (FIB) determination. Later at the time of euthanasia, after anesthesia with isoflurane, blood was drawn from the abdominal aorta of the animals scheduled for euthanasia in the primary test group for the determination of blood cell counts, coagulation function, and blood biochemical index. An automatic hematology analyzer, SYSMEX coagulometer, and TBA-120FR automatic biochemistry analyzer were used for the analysis.

### 2.11 Immunocyte phenotype analysis

For the primary test group (last 5 subjects per gender per group), 3 days post the last dosing (D18) and at the end of the recovery period (D32), blood was collected from the abdominal aorta after anesthesia with isoflurane (utilizing the same blood collection vessel as employed for blood cell count determination). The Beckman DxFLEX Flow Cytometry system was utilized for the analysis, with antibodies sourced from BioLegend. Both the staining procedure and subsequent analysis were meticulously conducted in compliance with the instructions outlined by the test kits. White blood cells were categorized using CD45, followed by demarcation of the lymphocyte population. The analysis assessed the percentages of peripheral blood total T lymphocytes (CD3+), Th cells (CD3+CD4+), and Tc cells (CD3+CD8+), and the ratio of CD3+CD4+/CD3+CD8+ was calculated.

### 2.12 Cytokine analysis

In the primary test group (equal gender proportion per group), on the third day subsequent to the last administration (D18) and at the end of the recovery interval (D32), blood samples were obtained from the abdominal aorta following isoflurane anesthesia, designated for cytokine assessment (utilizing the same blood collection vessel as employed for blood biochemistry analyses). The blood samples were maintained in an uncoagulated state, deposited in collection tubes embedded with separation gel and coagulant, ensuing in serum separation within a two-hour timeframe post-collection. The serum samples were stored under conditions below -20°C until the designated analysis. Utilizing the commercially accessible LEGENDplex™ Rat Assay Kit (Biolegend) in conjunction with the Beckman DxFLEX Flow Cytometry, the staining procedure and subsequent analysis were carried out in accordance with the instructions provided by the test kits, the content of tumor necrosis factor-α (TNF-α), interferon-γ (IFN-γ), interleukin-2 (IL-2), and interleukin-6 (IL-6) in serum were determined.

### 2.13 Analysis of serum antibody concentrations

Before each instance of drug administration (D-1, D14) and preceding the conclusion of the recovery period (D31), an approximate 2.0 ml of blood was drawn from the jugular vein of the animals in the satellite group. The collected blood samples were deposited in tubes containing separation gel and coagulant, centrifuged at 4°C, 3000×g for 10 minutes; following serum separation, the serum was apportioned into two tubes, each housing 250 μl, with the remaining serum relocated to a third tube. The serum samples were then stored at temperatures below -60°C. Utilizing the indirect ELISA method, the presence and concentration of anti-EM IgG antibodies in the serum were ascertained.

### 2.14 Autopsy and histopathological examination

The primary test group animals, designated for euthanasia on D18 and D32, were subjected to a systematic necropsy. Throughout the autopsy procedure, the animals’ body surface, body orifices, cranium, thoracic cavity, abdominal cavity, and their contents were inspected for any abnormalities. Tissue and organ fixation (eyeball, optic nerve, testes and epididymis were fixed in Davidson’s solution, while the remaining tissues/organs were stored in 10% neutral formalin) was carried out for histopathological examination.

### 2.15 Preclinical safety evaluation of EM

The preclinical safety evaluation of EM included single dose toxicity test, repeated dose toxicity test, active systemic allergy evaluation in guinea pigs and intradermal stimulation evaluation. (**Supplementary File S1**).

### 2.16 Statistical analysis

In present study, mean of skin test reactions were computed on SPSS 20. The two groups were compared with independent t- test and more than two groups were analyzed using one way Analysis of Variance (ANOVA). Single and repeated dosing of EM were analyzed on SAS 9.4 through ANOVA.

## 3 Results

### 3.1 Selection of recombinant MTB antigen

**Figure 1:**
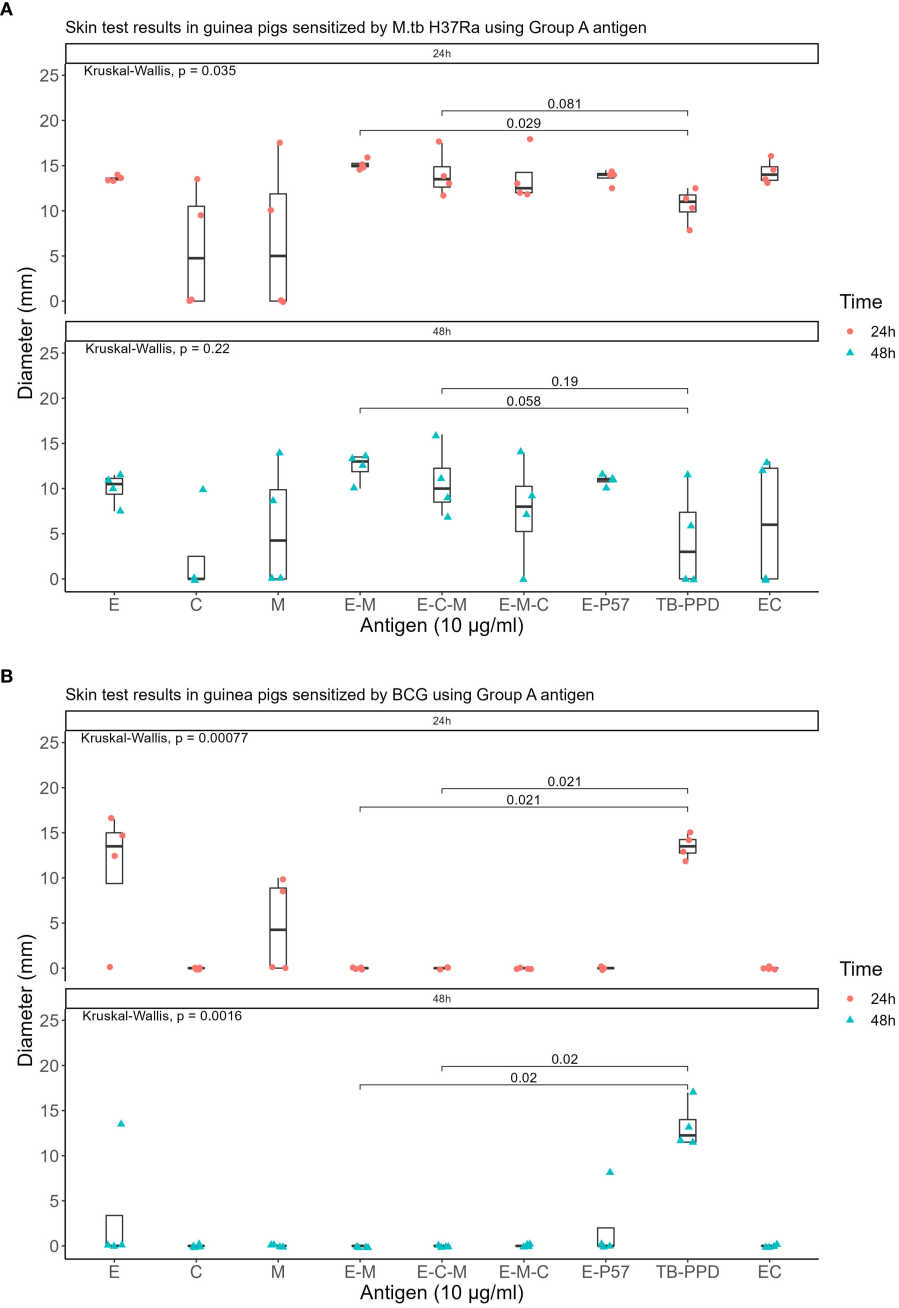
Skin test results at 10 μg/ml concentration within 24 and 48 hours using Group A antigens: **(A)** In guinea pigs sensitized by M.tb H37Ra **(B)** In guinea pigs sensitized by BCG.

**Figure 2:**
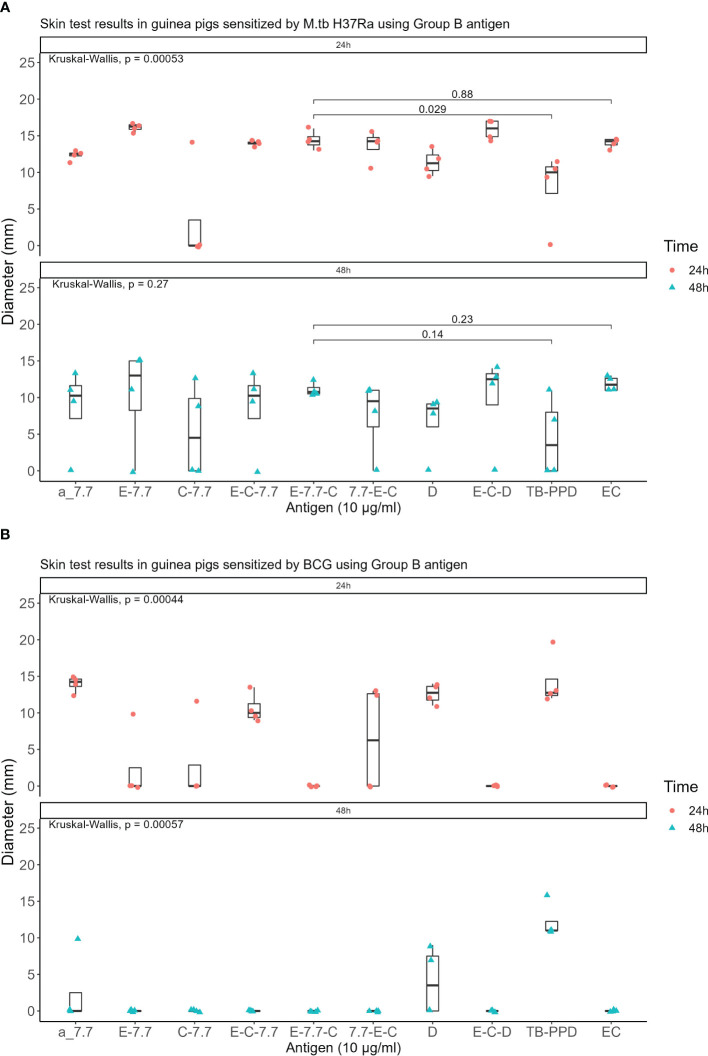
Skin test results using Group B antigen at 10 μg/ml concentration within 24 and 48 hours: **(A)** In guinea pigs sensitized by M.tb H37Ra **(B)** In guinea pigs sensitized by BCG.

**Figure 3:**
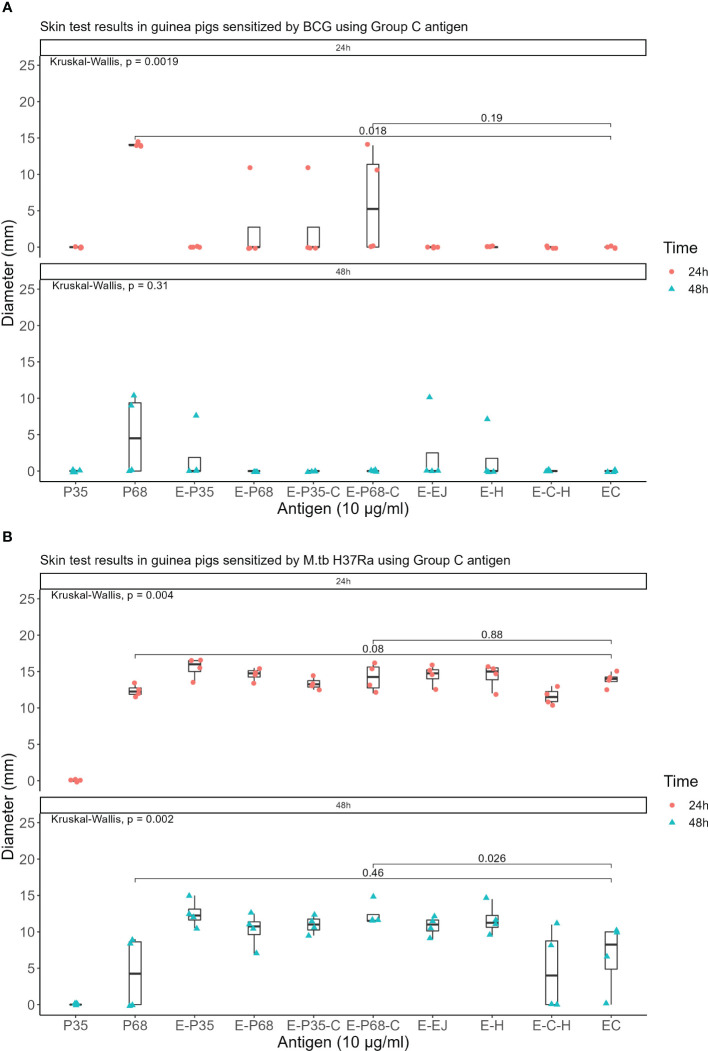
Skin test results using Group C antigen at 10 μg/ml concentration within 24 and 48 hours **(A)** In guinea pigs sensitized by M.tb H37Ra **(B)** In guinea pigs sensitized by BCG.

Seven recombinant MTB proteins (E, C, M, 7.7, D, PE35, and PPE68) alongside 17 fusion proteins (**Table 2**) were categorized into three groups (**Supplementary File S1**), and concomitantly underwent skin testing in both MTB and BCG sensitized guinea pigs. From group A, E-M and E-C-M (**Figure 1**), from Group B antigens, only E-7.7- (**Figure 2**) while none of the antigens from group C met the criteria for the second round of selection (**Figure 3**).

**Table 2:** Candidate proteins and their constructed recombinant fusion proteins.

No.	RD Region	Protein	Gene No.	Protein Name	Constructed Recombinant Fusion Proteins
1	RD1	PE35	Rv3872	P35	E-P57, E-M, E-C-M, E-M-C, E-7.7, C-7.7, E-7.7-C, E-7.7-C, 7.7-E-C, E-C-D, E-P35, E-P68, E-P35-C, E-P68-C, E-EJ, E-H, E-C-H
2	RD1	PPE68	Rv3873	P68	
3	RD1	CFP-10	Rv3874	C	
4	RD1	ESAT-6	Rv3875	E	
5	RD1	EspJ	Rv3878	EJ	
6	RD2	MPT64	Rv1980c	M	
7	RD11	PPE57	Rv3425	P57	
8	RD13	TB7.7	Rv2654c	7.7	
9	Non-RD region	HspX	Rv2031c	H	
10	Non-RD region	DPPD	Rv0061	D	

**Figure 4:**
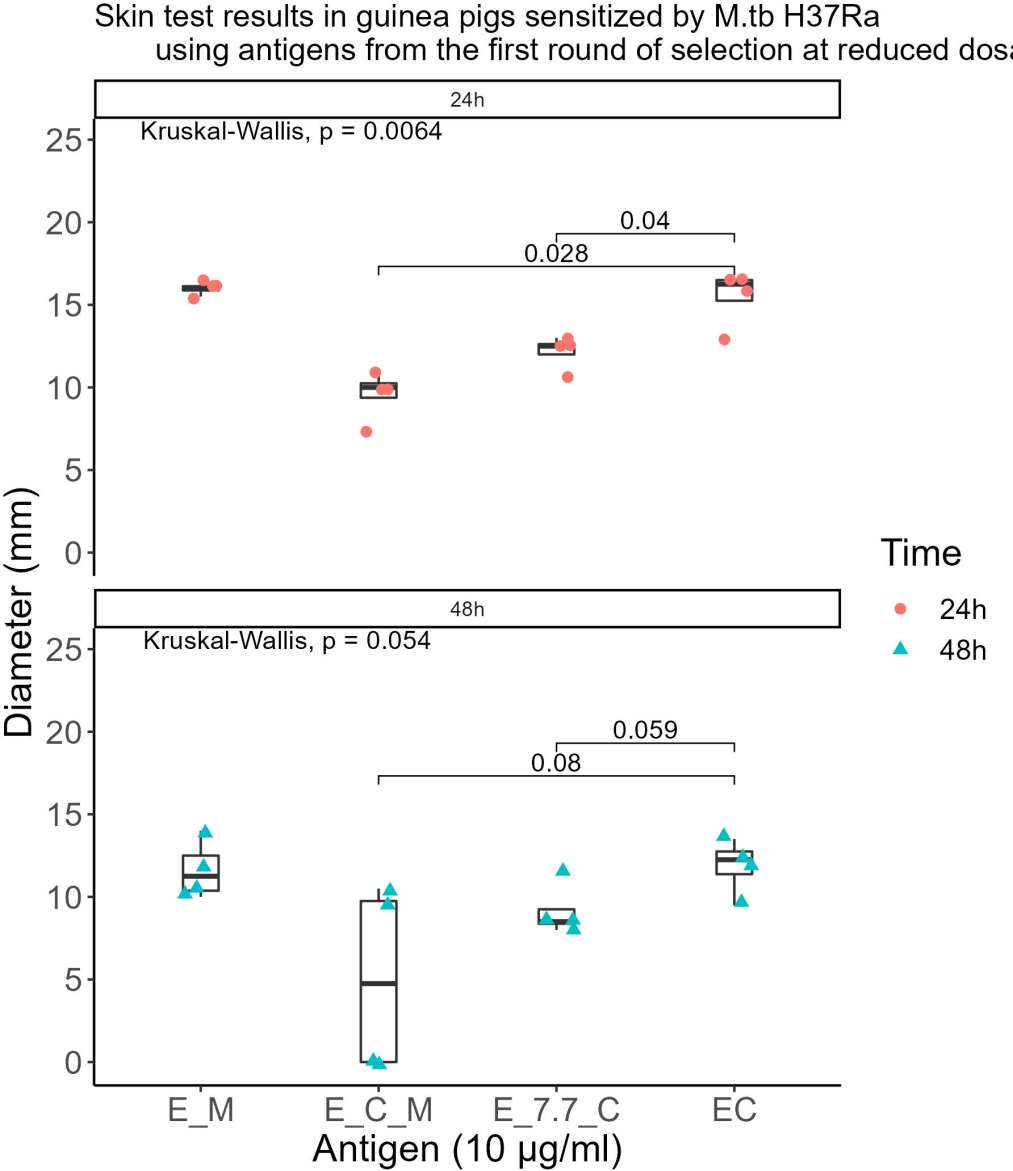
Skin test results in guinea pigs sensitized by M.tb H37Ra using antigens from the first round of selection at reduced dosages.

The qualified antigens were administered at a half dose for the skin testing in guinea pigs sensitized with MTB. At 24 hours post-administration, the mean skin reaction diameters in four guinea pigs for all selected antigens surpassed 5mm. The mean diameter for the skin reaction of E-M was not statistically significant (P>0.05) whereas E-C-M and E-7.7-C were significantly lower than those of the EC skin test reactions (P<0.001 and P<0.01 respectively) compared to that of the commercialized EC skin test reactions. At 48 hours, 50% of MTB-sensitized guinea pigs had reactions <5mm with E-C-M, and E-7.7-C reactions were significantly smaller than EC. E-M displayed similar intensity to EC without BCG cross-reactivity. (**Figure 4**).

### 3.2 Characteristics of EM skin test reactions

#### 3.2.1 EM-induced delayed-type hypersensitivity

**Figure 5:**
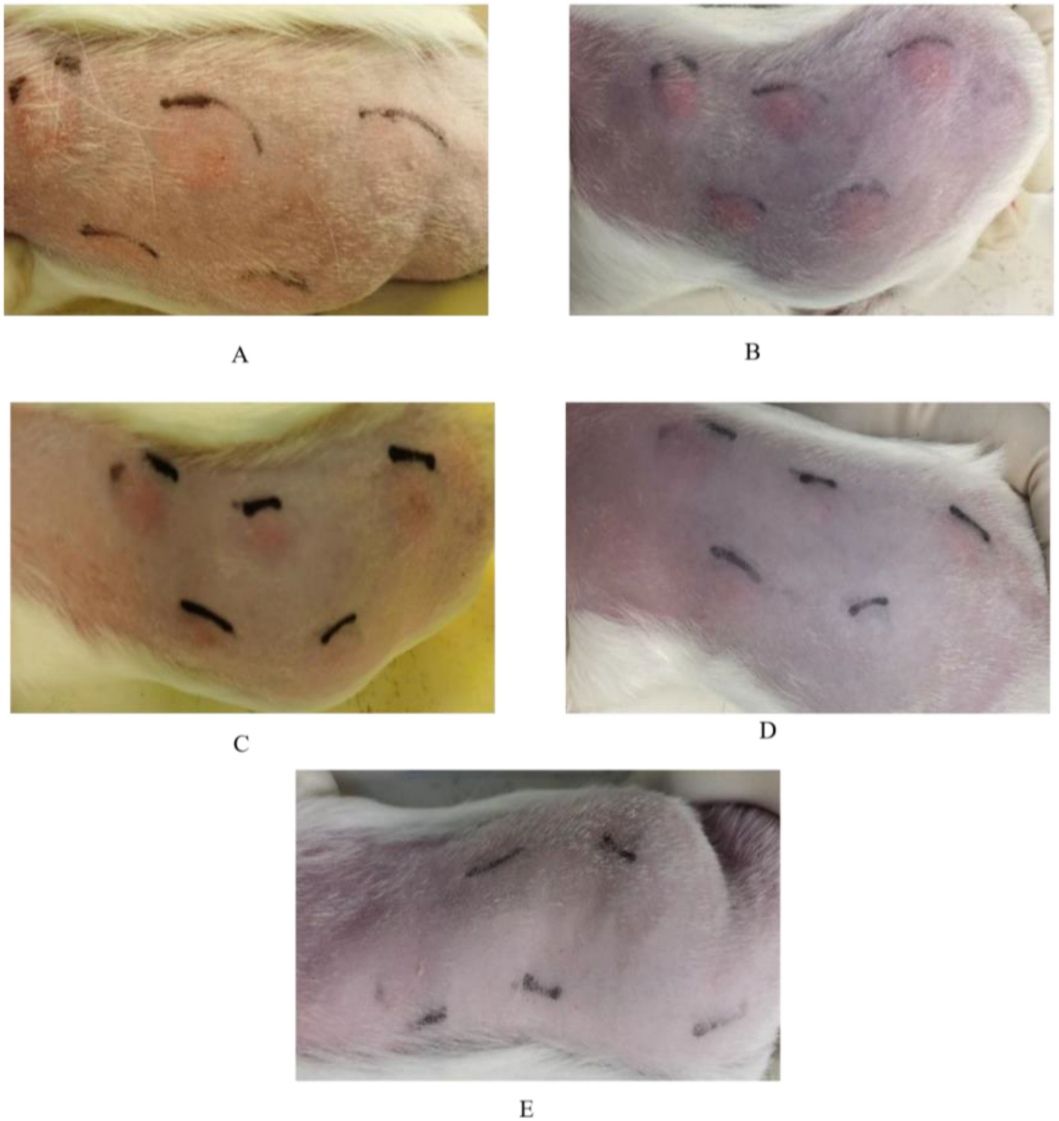
Changes in skin test reactions over time in guinea pigs sensitized by M.tb using different dosages of EM. **(A)** Skin test reaction 6 hours post-injection; **(B)** Skin test reaction 24 hours post-injection; **(C)** Skin test reaction 48 hours post-injection; **(D)** Skin test reaction 72 hours post-injection; **(E)** Skin test reaction 96 hours post-injection.

Different doses of EM, when tested on MTB-sensitized guinea pigs, shown skin test reactions at the injection site 6 hours post-injection; however, the boundaries of the reactions were unclear (**Table 3**, **Figure 5A**). At 24 hours post-injection, the reactions became more pronounced with clear boundaries (**Table 3**, **Figure 5B**). At 48 hours post-injection, there was little variation in reaction compared to the 24-hour reactions (**Table 3**, **Figure 5C**). By 72 hours post-injection, the reactions at the injection site weakened, with low-dose EM induced reactions mostly disappearing (**Table 3**, **Figure 5D**); and by 96 hours post-injection, all reactions at the injection site had disappeared (**Table 3**, **Figure 5E**).

**Table 3:** Variation in average diameter of skin test reactions induced by EM in M.tb-sensitized guinea pigs over time (unit: mm).

Skin test reagent	Average diameter of skin test reactions at different observation times						
	2h	4h	6h	24h	48h	72h	96h
TB-PPD	0	0	7.8 ± 2.0	13.0 ± 3.3	12.5 ± 2.4	8.5 ± 1.1	0
EC	0	0	12.5 ± 1.0	13.5 ± 4.3	12.6 ± 3.8	8.4 ± 4.9	0
10μg/ml EM	0	0	14.1 ± 1.0	18.3 ± 1.8	15.8 ± 1.3	6.9 ± 4.1	0
5μg/ml EM	0	0	12.7 ± 0.9	14.8 ± 1.3	13.3 ± 2.1	5.8 ± 4.9	0
2.5μg/ml EM	0	0	13.3 ± 1.3	15.1 ± 1.0	12.8 ± 1.6	3.5 ± 3.5	0

**Figure 6:**
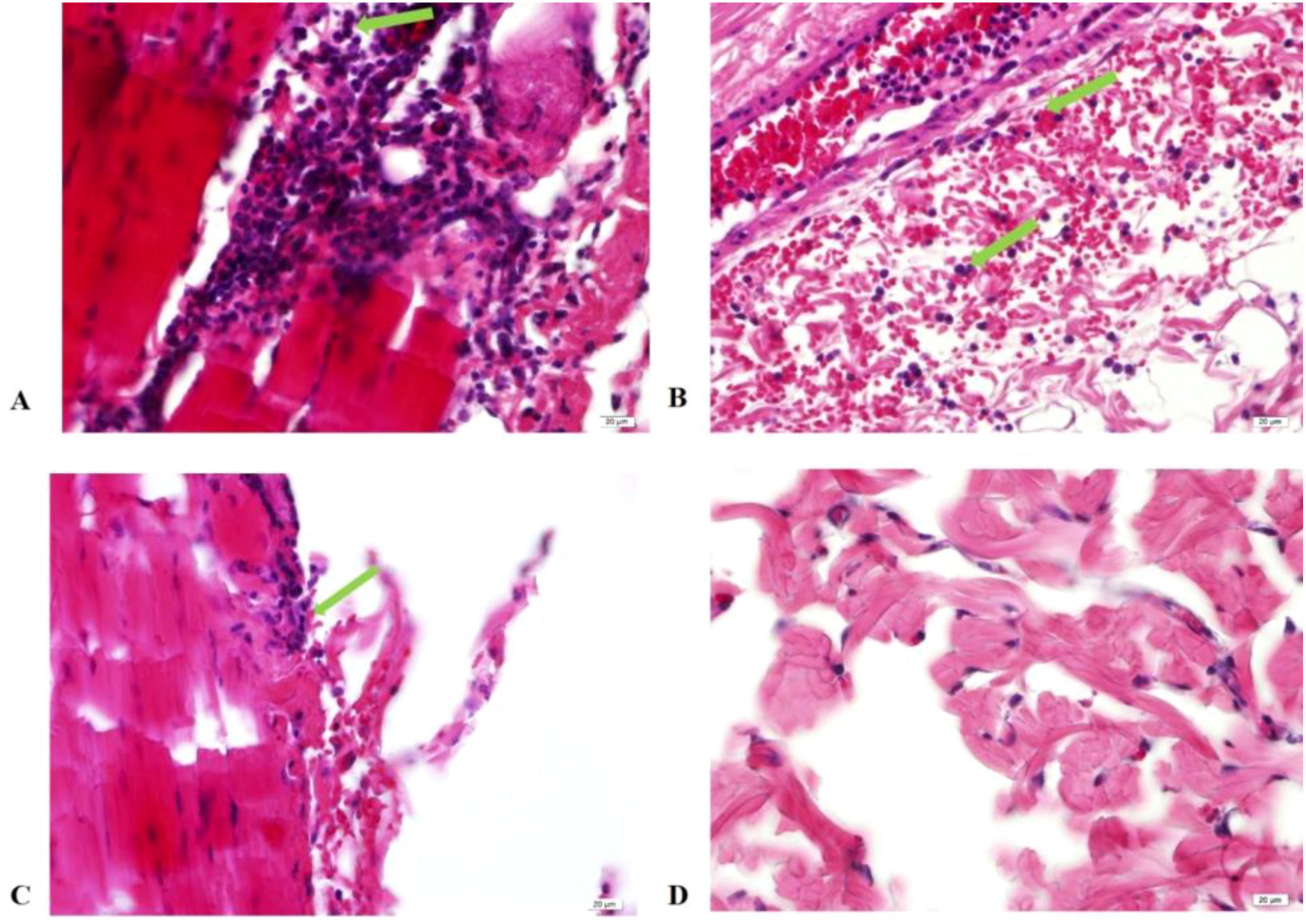
Histopathology of skin tissue at injection site 24 hours post-injection (HE×40). **(A)** Injection with EM; **(B)** Injection with EC; **(C)** Injection with TB-PPD; **(D)** Injection with solvent. Green arrows: Inflammatory cell infiltration visible in the dermis, subcutaneous fat tissue, and muscle layer.

At 24 hours post-skin test, histopathological examinations of the skin tissues at the injection sites of EM, EC, and TB-PPD were consistent, showing infiltrations of inflammatory cells, primarily lymphocytes with a higher nucleus-to-cytoplasm ratio, and a small number of neutrophils with segmented nuclei in the dermis, subcutaneous fat tissue, and muscle layer (**Figures 6A–C**); whereas the solvent injection sites displayed intact epidermal and dermal structures showing no apparent abnormalities (**Figure 6D**). Considering the onset time of EM skin test reactions and histopathological results from the injected skin site, EM reactions were categorized as delayed-type hypersensitivity (DTH) reactions.

#### 3.2.2 EM potency determination

A strong correlation coefficient R^2^ = 0.987 was observed between the cumulative value of the average diameters of 24-hour skin test reactions and the logarithmic value of the dose (log_10_) and R^2^ = 0.989 for 48-hour skin test reactions for EM at 2.5-10μg/ml concentration (**Supplementary Figure S1**).

#### 3.2.3 Specificity of EM skin test reactions

While considering the specificity of TB-PPD, PPD-B, EC, and EM for skin testing, it was observed that EM in MTB-sensitized, BCG-sensitized, inactivated MTB-sensitized, and NTM-sensitized guinea pig skin tests demonstrated comparable results to the marketed product EC with respect to other antigens; hence, EM mimics EC in specificity to MTB, devoid of BCG cross reactions (**Table 1**).

### 3.3 Preclinical safety evaluation results

#### 3.3.1 Single dose toxicity test

There was no statistically significant difference (P>0.05) in body weights, fatalities and signs of deterioration at different observation points pre- and post-administration between the low, high-dose EM groups, and the solvent control group (**Table 4**). Upon gross examination, no visible pathological lesions were found in any organs or at the drug injection sites of the skin.

**Table 4:** Body weights of rats in each group at different observation points before and after a single dose of EM (g).

Observation time	Male rats			Female rats		
	Solvent control	Low dose	High dose	Solvent control	Low dose	High dose
Pre-administration	310.4 ± 15.4	309.2 ± 17.4	306.2 ± 19.4	208.2 ± 11.4	206.6 ± 8.9	205.2 ± 7.9
7 days post-administration	353.0 ± 17.9	358.2 ± 21.3	346.2 ± 26.1	220.2 ± 15.9	222.0 ± 17.9	217.6 ± 10.0
14 days post-administration	398.8 ± 18.5	404.2 ± 26.0	387.2 ± 33.4	229.0 ± 11.0	228.0 ± 12.8	234.2 ± 9.2

Repeated dose toxicity test

Body weight and Body temperature: During the experiment period, the average body weights of the low- and high-dose EM administered groups were unaffected in comparison to the concurrent solvent control group at same instances (**Supplementary Table S1**). Minimal variations in average body temperatures of rats administered with different doses of EM at the mentioned time points were observed in comparison to the concurrent gender-matched solvent control groups, or simply occurred pre-medication. No discernible dose correlation or gender consistency was observed. Therefore, the changes in body temperature were deemed unrelated to EM administration (**Supplementary Table S2**).

Blood biochemistry: On days 4, 18, and 32 post-initial administration, various blood biochemistry parameters were assessed. Compared to the same-gender solvent control groups at the respective time points, no EM-related abnormalities were observed in any of the biochemical indices.

Urinalysis: All over the experiment period, no EM-related abnormalities were observed in urinalysis results of any groups when compared to both pre- and post-administration in same-gender solvent control groups at different time points.

Coagulation function: The coagulation function indices, including prothrombin time, activated partial thromboplastin time and fibrinogen, were assessed throughout the experiment period. Compared to concurrent same-gender solvent control groups, no EM-related abnormalities were observed in the cytokines function of any groups.

Patho-histology: Both gross and microscopic histopathological examinations revealed no EM-related changes or adverse reactions at the local injection sites on the skin with nil morbidity or moribund conditions.

Blood cell count: While comparing to concurrent same-gender solvent control groups, no EM-related abnormalities were observed in the blood cell counts of any groups at any instance of experimentation.

Immunotoxicology: At Day 18 and 32, the percentages of immunocytes in the blood of rats in both low and high dose EM groups were compared to the solvent control group, with no statistically significant differences noted (P>0.05) (**Supplementary Table S3**). No EM-related abnormalities spotted in the cytokines of each dosing group when compared with the same-gender solvent control group of the same period.

The results of serum antibody determination on Day-4 (pre-administration), Day 14 (pre-second administration), and Day 31 (pre-end of recovery period) are shown in **Supplementary Table S4**.

### 3.4 Active systemic allergy test

At 14 days post-last sensitization, all guinea pigs in the positive control group exhibited positive reactions (++) upon challenge. The responses of guinea pigs in the different dosed EM groups were comparable to those in the negative control group (**Supplementary Table S5**).

### 3.5 Intradermal stimulation test

Overall, no abnormal reactions were noted at the administration sites during local clinical observations. All rabbits exhibited normal weight gain, with no mortality or near-death situations.

Animals euthanized on Day 3 post-administration (D4) and Day 14 post-administration (D15) showed no noticeable abnormal changes upon gross anatomical examination of the administration sites. On Day 4 histopathological examination of the administration sites in animals given sodium chloride injection, solvent, low-dose EM, and high-dose EM displayed inconspicuous abnormal changes (**Supplementary Figure S2**). Hence, the solitary instance of local pathological change in the animals administered with solvent control was deemed incidental and unrelated to the solvent control.

## 4 Discussion

BCG vaccination and NTM infection reduce the specificity of TB-PPD skin testing to diagnosis MTB infection. Utilizing MTB-specific, BCG-depleted antigens as a substitute for TB-PPD has proven to enhance the specificity of tuberculin skin testing. Three new highly specific tuberculin skin testing diagnostic reagent products, formulated with BCG-depleted antigens ESAT-6 and CFP-10 in MTB RD1 region, have become available in market (21, 22) for tuberculosis diagnosis. WHO has designated and recommended the recombinant MTB antigen skin testing as the most appropriate TBST method for MTB infection screening in 2022 (23).

Globally, all the TBST method based commercially available products consist of recombinant or fusion proteins of ESAT-6 and CFP-10 (11). These advances are encouraging, as in China BCG vaccination is prevalent and TB-PPD shares a substantial number of cross-reactive antigens with BCG, obscuring the MTB infection and BCG vaccination distinction. Therefore, the novel skin test antigens for MTB should have no cross-reactivity with BCG, possibly achieved by BCG-depleted antigens employment (24). To develop new tuberculin skin testing diagnostic reagents based TBST method, 24 recombinant MTB antigens were evaluated within the same system. Initially, 0.1 ml of 10μg/ml of recombinant MTB antigens were simultaneously administered to MTB sensitized guinea pigs and BCG sensitized guinea pigs. Antigens E-M, E-C-M, and E-7.7-C met the criteria for the next round with a reduced dosage (5μg/ml) for skin testing in MTB sensitized guinea pigs. Only the skin test reaction intensity of E-M was comparable to the marketed product EC. Reportedly, all BCG vaccine strains, except those derived from BCG Pasteur strain before 1925 (like Brazilian, Japanese and Russian BCG sub-strains), lost the MPT64 protein-encoding gene *Rv1980c* in their genomes. MPT64 (25) can differentiate MTB infection from NTM infection (26). MPT64, when combined with ESAT-6, low-dose E-M can induce a skin test reaction intensity in MTB sensitized guinea pigs comparable to the marketed product EC. Ultimately, E-M was selected for the development of tuberculin antigens for skin testing.

The utilization of E-M based tuberculin antigens for skin testing aimed towards clinical application. Given that the current selected E-M is composed of tag-purified tuberculin antigens, there may be safety risks for clinical purposes due to the incomplete SUMO tag removal.

Hence, a recombinant untagged E-M protein expression vector should be reconstructed for production following GMP standards. Quality-approved EM should then be employed for the study of skin test reaction profiles and specificity in pre-clinical safety assessment and potency evaluations.

Specificity studies indicated that the BCG-sensitized guinea pigs, NTM-sensitized guinea pigs, and inactivated MTB sensitized guinea pigs exhibited comparable skin test reactions to the selected MTB antigen (EM) and the marketed product EC (6). Specifically, BCG-sensitized guinea pigs, various tested NTM strains (excluding *Mycobacterium kansasii*), and inactivated MTB sensitized guinea pigs all exhibited negative reactions to both skin tests antigens. Literature has reported, BCG Danish 1331 and avian mycobacteria sensitized guinea pigs both showed negative skin reactions to recombinant ESAT-6 protein and recombinant MPT64 protein, but positive reactions to TB-PPD (27). Our experimental and literature findings suggested that ESAT-6 and MPT64 have shown no cross-reactivity with the Chinese BCG vaccine strain, BCG Danish 1331 and most NTM strains. Therefore, it is proposed that EM will be able to effectively differentiate between MTB infection and BCG vaccination/NTM infection.

In the case of MTB sensitized guinea pigs, skin reactions appeared at the administration site 6 hours after EM injection, reaching peak intensity at 24 hours, which was easily observable. The histological presentations of the skin at the 24-hour post-administration site were consistent with those seen with TB-PPD, both exhibiting characteristics of DTH reactions. Hence, the skin reactions to EM in MTB sensitized guinea pigs were indicative of DTH responses. Potency determination of EM indicated that the skin test reaction intensity induced by 2.5-10μg/ml EM was linearly correlated with dosage. The preclinical safety evaluation of the selected MTB antigen (EM) for skin testing, showed no abnormal reactions related to the administration.

We demonstrated EM as a promising candidate for the development of a tuberculin antigen skin test. Current study got some limitations which need to be addressed for better preclinical evaluation outcome in near future. Firstly, due to the safety concerns poses by potential contamination of uncleaved SUMO tagged recombinant protein used to generate the untagged EM in this study, future preclinical evaluation should be done using untagged EM under Good Manufacturing Practice (GMP) conditions.

The product compatibility between preclinical and clinical trials is mandatory to ensure its purity to address the safety concerns. Secondly, model animals used for the present study depicted no abnormal reaction as they are less related to humans in comparison to non-human primates (NHPs) in physiology and immunology. Hence, exploring the application of EM in NHPs could provide more relevant data. At large, as far as the immunogenicity in animal models is concerned, some rats developed anti-EM-IgG antibodies, particularly at high doses. These immunogenic responses does not always predict human responses, therefore, E-M’s immunogenic potential in humans is yet to explore. Addressing all these limitations will be helpful for the successful declaration of EM as a tuberculin antigen skin test in future research.

In conclusion, MTB antigen (EM) for skin testing demonstrated safety and efficacy at preclinical level. As EM exhibited successful specificity for MTB detection of tuberculosis infection, it is highly recommended for clinical trial to enhance MTB diagnosis leading to efficient treatment. Further applications for clinical research are warranted to assess the clinical utility of EM in the diagnostic approach to tuberculosis. During clinical trials, exclusive monitoring for adverse reactions at the injection site, like blistering or ulceration, is recommended. Insights from parallel products, such as EC, should be focused for monitoring adverse events during EM trials.

## Data Availability

The datasets presented in this study can be found in online repositories. The names of the repository/repositories and accession number(s) can be found below: https://www.ncbi.nlm.nih.gov/genbank/, PP993260.
