# Supplementary material for: Screening and preclinical assessment of novel Mycobacterium tuberculosis recombinant antigens based tuberculin skin testing
Source: Front Immunol. 2025 Mar 7;16:1498448. doi: 10.3389/fimmu.2025.1498448 (PMC11925772; doi:10.3389/fimmu.2025.1498448)
Supplement: Supplementary file 1 [file SupplementaryFile1.docx]

**Supplementary Material S1 Preclinical safety evaluation of EM**

**Single dose toxicity test**

The study utilized 30 SPF-grade SD rats aged 7 to 9 weeks, with an equal proportion of males and females. The animals were divided into three groups: high dose, low dose, and solvent control, each containing 10 SD rats (5 males and 5 females). The high dose group received an intradermal injection of EM at 5 μg/0.1 ml per rat while the low dose group was administered EM at 0.5 μg/0.1 ml per rat. The solvent control group was administered 0.1 ml of an EM diluent. Each group was observed prior to dosing and continuously for 4 hours post-dosing, followed by twice daily for 14 consecutive days, to highlight any mortalities or moribund state. The injection site was examined for signs like erythema, congestion, swelling, ulceration, and induration a day before dosing, the day after, and once weekly thereafter. Rats were weighed before the treatment, then on days 7 and 14 post-treatment. After 14 days of observation, euthanasia was carried out with all rats by CO_2_ inhalation, and death was confirmed via femoral artery bleeding. A gross necropsy was performed on all the animals, looking for external abnormalities, orifices, cranial, thoracic, and abdominal cavities, including their contents, and checking organs and injection sites for abnormal pathological changes.

**Repeated dose toxicity test**

The test involved 120 SPF-grade SD rats, aged 6 to 7 weeks, split equally between males and females. These were divided into primary test groups and satellite groups. The primary test groups comprised the high dose, low dose, and solvent control sub-groups, each with 30 SD rats (15 males and 15 females). Similarly, the satellite groups encompassed the high-dose, low-dose, and solvent control sub-groups, each containing 10 SD rats, again with an equal gender distribution. High dose groups were intradermally injected with 5 μg/0.1 ml EM. In the low-dose groups, each rat was administered EM at 0.5 μg/0.1 ml. The solvent control groups were given an intradermal injection of 0.1 ml EM diluent. The initial dosing day was labeled as D1, and another dose was administered on D15 (two doses). The very next day following the last dose was nominated as the first recovery day. All animals were observed for clinical observations; weighing; body temperature measurement; blood cell count, coagulation function, and blood biochemistry analysis; urine analysis and Immunocyte phenotype analysis and autopsy and histopathological examination: The primary test group animals, designated for euthanasia on D18 and D32, were subjected to a systematic necropsy. Throughout the autopsy procedure, the animals’ body surface, body orifices, cranium, thoracic cavity, abdominal cavity, and their contents were inspected for any abnormalities. Tissue and organ fixation (eyeball, optic nerve, testes and epididymis were fixed in Davidson’s solution, while the remaining tissues/organs were stored in 10% neutral formalin) was carried out for histopathological examination.

**Intradermal stimulation evaluation**

A total of eight New Zealand rabbits were subjected to the self-control method, receiving intradermal injections of 0.1 ml sodium chloride solution, 0.5 μg EM, 1 μg EM, and solvent on both dorsal sides, maintaining a minimum of 5 cm distance between any two injection points. All subjects were observed at least once respectively during the morning and afternoon daily throughout the experimentation period, checking for mortality, clinical manifestations, and excreta.

Local observation post-administration: Observations were conducted once before and once after administration on the administration day, and once daily during subsequent times. On day 3 post-administration (D4) (local observation timeframe post-administration was 72 ±2 hours), any visible and palpable lesions were observed, including erythema, congestion, swelling, ulceration, and induration, etc.

At 72 hours post-administration, five rabbits were euthanized; the remaining three rabbits were euthanized on day 14 post-administration. Following a gross observation of the local tissue post-administration, the tissues were sampled and fixed in 10% neutral formalin for subsequent histopathological examination.
