# Supplementary material for: Screening and preclinical assessment of novel Mycobacterium tuberculosis recombinant antigens based tuberculin skin testing
Source: Front Immunol. 2025 Mar 7;16:1498448. doi: 10.3389/fimmu.2025.1498448 (PMC11925772; doi:10.3389/fimmu.2025.1498448)
Supplement: Supplementary file 2 [file SupplementaryFile2.docx]

**Supplementary Material 2**

**S2 Table 1: Body weights of rats in each group at different observation points before and after repeated doses of EM (g)**

| Observation time | Male rats | | | Female rats | | |
| --- | --- | --- | --- | --- | --- | --- |
|  | Solvent control group | Low dose group | High dose group | Solvent control group | Low dose group | High dose group |
| Pre-administration | 231.5±7.9 | 232.3±8.3 | 232.2±8.3 | 202.9±7.2 | 202.9±6.6 | 202.7±6.7 |
| 2 days post-initial administration | 243.7±8.8 | 245.0±8.8 | 245.3±9.7 | 210.3±8.3 | 210.7±9.3 | 212.8±6.9 |
| 4 days post-initial administration | 260.8±9.7 | 261.7±12.0 | 262.9±10.4 | 219.9±8.9 | 215.3±8.2 | 217.1±8.3 |
| 7 days post-initial administration | 287.0±10.5 | 288.2±14.4 | 287.3±15.1 | 232.5±11.5 | 228.9±9.5 | 231.3±11.3 |
| 14 days post-initial administration | 343.5±15.3 | 340.4±22.8 | 342.7±24.4 | 252.2±17.7 | 249.1±13.5 | 258.9±16.5 |
| 18 days post-initial administration | 343.1±13.2 | 347.7±21.1 | 341.8±23.1 | 238.9±14.1 | 241.7±12.5 | 250.1±11.7 |
| 21 days post-initial administration | 373.8±15.4 | 347.0±21.4 | 366.4±36.8 | 270.8±7.3 | 264.2±7.6 | 262.6±8.5 |
| 28 days post-initial administration | 410.4±15.6 | 383.0±28.9 | 397.2±45.8 | 274.0±7.8 | 271.0±13.2 | 274.4±18.7 |
| 32 days post-initial administration | 401.4±16.7 | 377.6±24.4 | 387.6±46.3 | 267.8±10.8 | 262.4±9.5 | 261.2±16.3 |

**S2 Table 2: Body temperatures of rats in different groups at various observation points before and after repeated doses of EM (℃)**

| Observation time | Male rats | | | Female rats | | |
| --- | --- | --- | --- | --- | --- | --- |
|  | Solvent control | Low dose | High dose | Solvent control | Low dose | High dose |
| Pre-administration | 37.04±0.36 | 37.34±0.30 | 37.72±0.31* | 37.88±0.43 | 37.64±0.34 | 37.58±0.58 |
| 1day post-initial administration | 37.14±0.17 | 37.62±0.11* | 37.10±0.17 | 38.14±0.62 | 37.74±0.56 | 37.24±0.25* |
| 2 days post-initial administration | 36.32±0.25 | 36.70±0.55 | 37.16±1.02 | 37.08±0.76 | 37.28±0.43 | 38.24±0.63* |
| 15 days post-initial administration | 36.28±0.16 | 36.28±0.30 | 37.10±0.41* | 38.40±0.31 | 38.56±0.41 | 38.70±0.55 |
| 16 days post-initial administration | 37.14±0.15 | 36.90±0.20 | 37.22±0.08 | 38.24±0.37 | 37.84±0.59 | 38.34±0.57 |
| 31 days post-initial administration | 37.50±0.38 | 36.53±0.16* | 37.44±0.19 | 38.32±0.28 | 38.28±0.30 | 38.14±0.29 |

*: Indicates statistically significant difference compared to the same-gender solvent control group (*P<*0.05).

**S2 Table 3: Percentages of immunocytes in blood samples collected at different time points post-EM repeated dose**

| Date of determination | Group | CD3+% | CD3+CD4+% | CD3+CD8+% | CD3+CD4+/  CD3+CD8+ |
| --- | --- | --- | --- | --- | --- |
| 3 days post-last administration (D18) | Solvent control | 45.53±8.41 | 61.81±5.66 | 35.28±6.45 | 1.86±0.58 |
|  | Low dose | 45.51±10.33 | 62.39±5.39 | 34.72±6.24 | 1.90±0.56 |
|  | High dose | 46.13±10.48 | 61.61±6.34 | 33.36±8.89 | 2.06±0.82 |
| End of the recovery period (D32) | Solvent control | 54.67±8.10 | 68.90±5.82 | 29.15±5.86 | 2.59±1.16 |
|  | Low dose | 49.81±6.93 | 67.00±5.76 | 30.80±5.90 | 2.32±0.76 |
|  | High dose | 53.58±6.09 | 67.42±6.24 | 30.50±6.22 | 2.40±0.99 |

**S2 Table 4: Serum anti-EM-IgG antibody determination results at different time points post-EM repeated dose**

| Blood collection time | Group | Dose | Male | | Female | |
| --- | --- | --- | --- | --- | --- | --- |
|  |  | **(μg/animal)** | **Antibody positivity rate** | **Antibody titer range** | **Antibody positivity rate** | **Antibody titer range** |
| D-4 | Low dose | 0.5 | 0/5 | / | 0/5 | / |
|  | High dose | 5 | 0/5 | / | 0/5 | / |
| D14 | Low dos | 0.5 | 0/5 | / | 0/5 | / |
|  | High dos | 5 | 1/5 | 1:40 | 2/5 | 1:20-1:320 |
| D31 | Low dose | 0.5 | 0/5 | / | 1/5 | 1:320 |
|  | High dose | 5 | 0/5 | / | 3/5 | 1:40-1:40960 |

Note: “/” indicates that titer determination was not performed. The results of the antibody positivity rate are presented as “Number of animals with positive antibodies/Number of animals tested”.

**S2 Table 5: Responses of guinea pigs sensitized with different doses of EM at different challenge time points**

| Group | Challenge time | Number of guinea pigs showing allergic reactions | Number of dead guinea pigs | Reaction level | Type of allergic reaction |
| --- | --- | --- | --- | --- | --- |
| Negative control | D19 | 0 | 0 | - | Negative |
|  | D26 | 0 | 0 | - | Negative |
| Low dose EM | D19 | 0 | 0 | - | Negative |
|  | D26 | 0 | 0 | - | Negative |
| High dose EM | D19 | 0 | 0 | - | Negative |
|  | D26 | 0 | 0 | - | Negative |
| Positive control | D19 | 3 | 0 | ++ | Negative |
|  | D26 | 6 | 3 | +++ and ++++ | Strong positive (3) / Extremely strong positive (3) |

**Supplementary Material 2**

**Figures:**


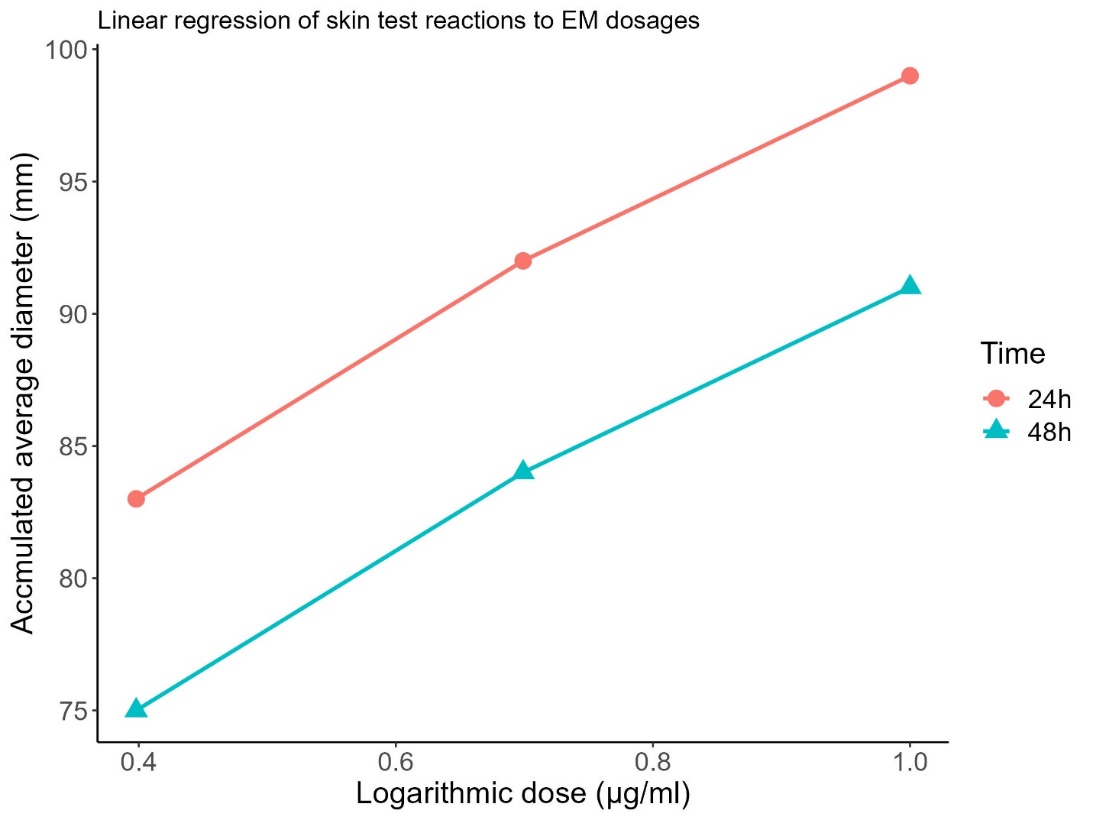


**S2 Figure 1: Linear regression of skin test reactions to EM dosages at 24 and 48 hours**


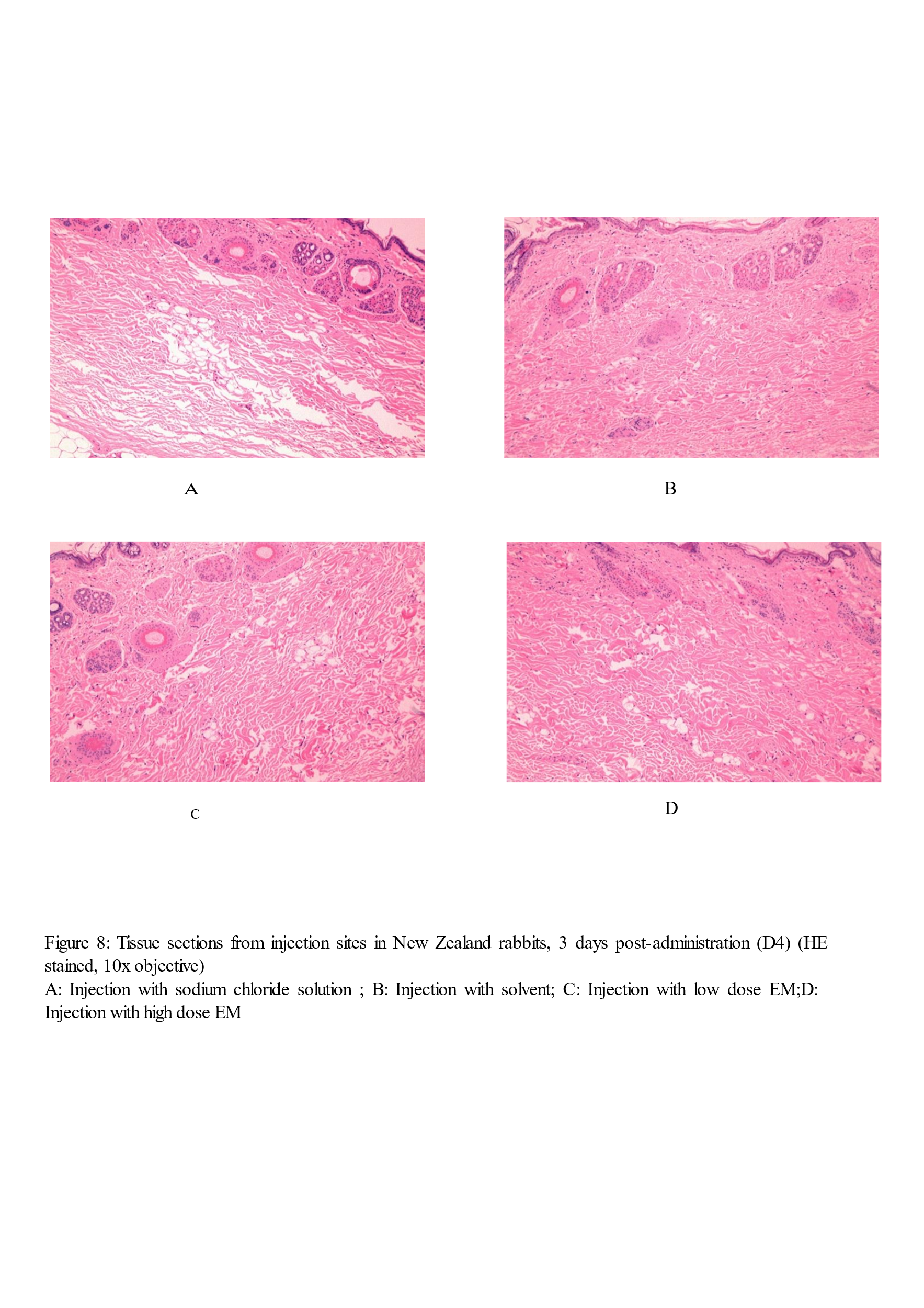


**S2 Figure 2: Tissue sections from injection sites in New Zealand rabbits, 3 days post-administration (D4) (HE×10**)**. A: Injection with sodium chloride solution; B: Injection with solvent; C: Injection with low dose EM; D: Injection with high dose EM.**
