# Supplementary material for: Screening and preclinical assessment of novel Mycobacterium tuberculosis recombinant antigens based tuberculin skin testing
Source: Front Immunol. 2025 Mar 7;16:1498448. doi: 10.3389/fimmu.2025.1498448 (PMC11925772; doi:10.3389/fimmu.2025.1498448)
Supplement: Supplementary file 3 [file SupplementaryFile3.docx]

Supplementary file 1. The nucleotide sequence encoding the recombinant antigen E-M.

MTEQQWNFAGIEAAASAIQGNVTSIHSLLDEGKQSLTKLAAAWGGSGSEAYQGVQQKWDATATELNNALQNLARTISEAGQAMASTEGNVTGMFAGGGSGGGGSGMAPKTYCEELKGTDTGQACQIQMSDPAYNINISLPSYYPDQKSLENYIAQTRDKFLSAATSSTPREAPYELNITSATYQSAIPPRGTQAVVLKVYQNAGGTHPTTTYKAFDWDQAYRKPITYDTLWQADTDPLPVVFPIVQGELSKQTGQQVSIAPNAGLDPVNYQNFAVTNDGVIFFFNPGELLPEAAGPTQVLVPRSAIDSMLA
