# Supplementary figures and images for: Screening and preclinical assessment of novel Mycobacterium tuberculosis recombinant antigens based tuberculin skin testing
Source: Front Immunol. 2025 Mar 7;16:1498448. doi: 10.3389/fimmu.2025.1498448 (PMC11925772; doi:10.3389/fimmu.2025.1498448)

S4. SDS-PAGE reducing gel. 3 ug of sample loaded to each well.


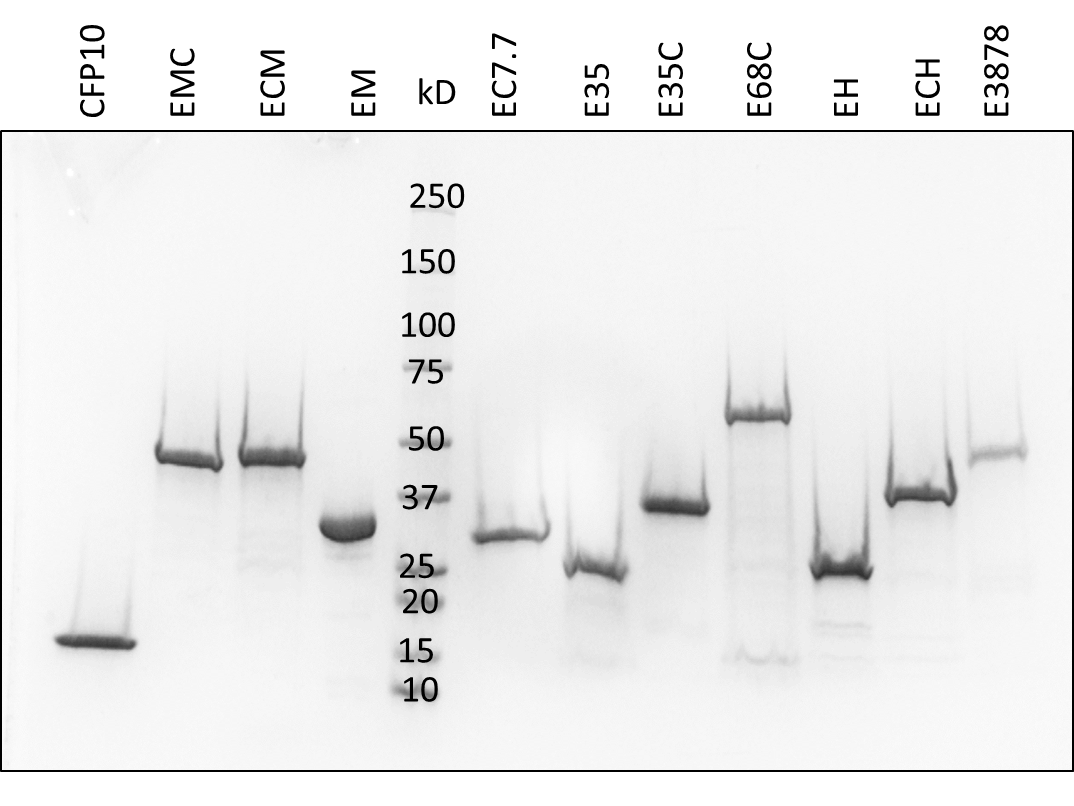


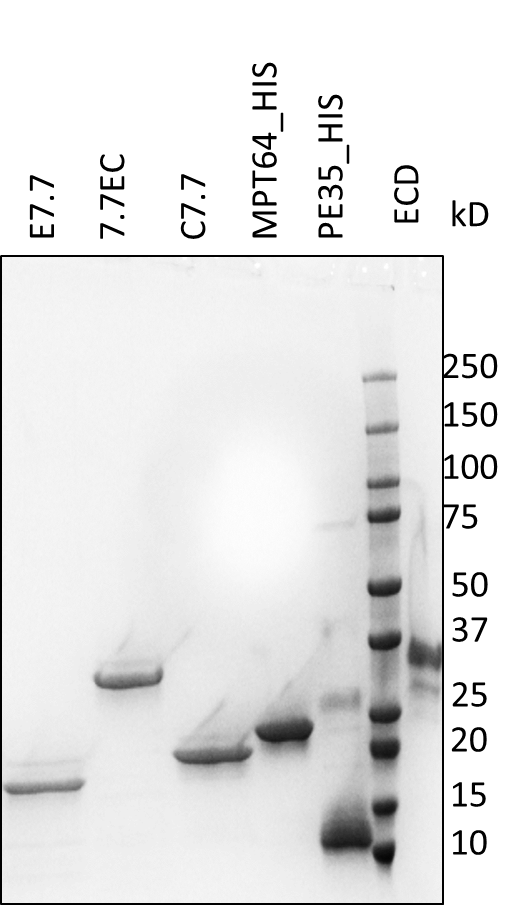

Supplement: Supplementary file 4 [file SupplementaryFile4.docx]
